# Patterns of Occurrence and Outcomes of Contralateral Breast Cancer: Analysis of SEER Data

**DOI:** 10.3390/jcm7060133

**Published:** 2018-05-31

**Authors:** Zhenchong Xiong, Lin Yang, Guangzheng Deng, Xinjian Huang, Xing Li, Xinhua Xie, Jin Wang, Zeyu Shuang, Xi Wang

**Affiliations:** 1 Department of Breast Surgery, State Key Laboratory of Oncology in Southern China, Collaborative Innovation Center for Cancer Medicine, Sun Yat-sen University Cancer Center, Guangzhou 510030, China; xiongzhch@sysucc.org.cn (Z.X.); denggzh@sysucc.org.cn (G.D.); huangxj1@sysucc.org.cn (X.H.); lixing@sysucc.org.cn (X.L.); xiexh@sysucc.org.cn (X.X.); wangjin1@sysucc.org.cn (J.W.); shuangzy@sysucc.org.cn (Z.S.); 2 State Key Laboratory of Oncology in Southern China, Collaborative Innovation Center for Cancer Medicine, Sun Yat-sen University Cancer Center, Guangzhou 510030, China; yanglin@sysucc.org.cn

**Keywords:** contralateral breast cancer, incidence, outcome, SEER, propensity score

## Abstract

Population-based estimates are lacking for the temporal trends in the contralateral breast cancer (CBC) risk for patients with breast cancer (BC). Data for BC patients diagnosed with CBC were collected from the Surveillance, Epidemiology, and End Results database. CBC incidence was calculated using the Kaplan-Meier method and the temporal trend in CBC incidence was assessed using joinpoint regression. Survival analysis was calculated using propensity scoring (PS) and multivariate Cox regression with a competing risk model. We found that 10,944 of 212,630 patients with early-stage BC were subsequently diagnosed with secondary BC in the contralateral breast. The 5-, 10-, 15-, and 20-year cumulative CBC incidences were 1.9, 4.6, 7.6, and 10.5%, respectively. Being younger (<40 years), black, hormone receptor-negative, and having undergone radiotherapy were correlated with a high risk of CBC occurrence. CBC incidence increased continuously in the first 11 years after the initial cancer diagnosis, and the upward trend slowed from years 11 to 21, and tended to decline from years 21 to 24. CBC diagnosis was significantly and negatively associated with survival. We reported population-based estimates of the CBC occurrence pattern and risk factors. Patients are at high risk of developing CBC in the first 21 years after the initial BC diagnosis.

## 1. Introduction

The incidence of contralateral breast cancer (CBC) is about 6.1% 10 years after the initial diagnosis of breast cancer (BC) [1,2,3]. Although previous studies showed a favorable decrease in CBC incidence, improved survival, increased incidence, and decreased patient age have led to an increasing number of BC patients at risk of developing contralateral secondary BC [1,2,4].Therefore, surveillance of metachronous CBC in patients with BC is important.

The risk of BC is higher in patients with BC compared to the risk in the general population, whereas the variation in the trend of CBC incidence, which is the change in CBC incidence per year during the follow-up in BC patients, has been seldom reported [5,6,7]. Moreover, initial cancer treatment may affect CBC risk, where endocrine treatment and chemotherapy can reduce CBC risk and delay CBC development [8,9,10]. This renders the occurrence pattern of CBC unpredictable; therefore, optimal surveillance of CBC becomes challenging for cancer diagnoses. Another challenge faced by CBC surveillance is that the population of BC patients with high CBC risk is unknown, and studies on the risk factors of CBC are limited. Patients with *BRCA1* or *BRCA2* gene mutation or with tumor history at a young age (<35 years) have significantly higher CBC risk, but represent only a small proportion of the CBC population [11,12,13]. Identifying the patient and tumor characteristics corresponding to high CBC risk would be helpful for efficient surveillance of patients with BC.

Previous studies reported that the early development of CBC is associated with worse survival [4,14]. Post-therapy surveillance is of considerable value for BC patients to discover CBC in the early stages to improve survival. Thus, patients with BC should receive periodic follow-up, including physical and imaging examination [15]. Studies of CBC occurrence regularity is important to guide the formulation of a surveillance plan for patients with BC. 

In this study, we used the National Cancer Institute’s Surveillance, Epidemiology, and End Results (SEER) database to estimate the CBC occurrence pattern in BC and to identify the population of BC patients who are susceptible to CBC. Moreover, we compared the overall survival (OS) and BC-specific survival of patients with CBC to that of patients with unilateral disease in early-stage BC.

## 2. Experimental Section

### 2.1. Patients and Methods

Patient information was collected from the SEER database, which includes nine registries [16]. We defined BC with T_1–2_, N_0–1_, M_0_ as early-stage BC, and included patients with no history of cancer who were diagnosed with BC between January 1990 and December 2009. CBC is defined as an invasive breast cancer diagnosed in the contralateral breast 2 months or more after the first diagnosis of invasive BC. Patients with local advanced or initial distant metastatic BC were not included, due to the poor survival of this group, as most of them may die of cancer progression before the CBC occurs. We excluded patients with: (1) diagnosis only on death certificate or autopsy; (2) follow-up <2 months; (3) unknown age or <18 years; (4) synchronous bilateral breast cancer; (5) or prophylactic removal of the contralateral breast, leaving 212,630 patients in the final study population. Among them, 10,944 patients were subsequently diagnosed with CBC. The follow-up period was from January 1990 to December 2013.

### 2.2. Statistical Analysis

We calculated the cumulative incidence of CBC using the Kaplan-Meier method [17]. CBC incidence was recorded per 1000 person-years. We then used joinpoint regression to analyze trends of CBC incidence and mortality risk. Joinpoint regression involves straight-line segment fitting to the cumulative incidence of CBC, which are intersected at the joinpoint, where significant changes in trend occur. Rate changes were assessed using annual percent change (APC). Standardized incidence ratio (SIR), a ratio of observed to expected incidence based on the general population rates, was used to assess excess relative risk of CBC compared with the general population. Univariate and multivariate Cox regression models were used to identify CBC risk factors.

Overall survival (OS) was calculated from the date of the latest diagnosis of BC to the date of death. As our study was retrospective, selection bias may have occurred; therefore, multivariate analysis and the propensity score (PS) model were used to adjust for potential confounding factors from the following variables: age, Hispanic origin, race, sex, year of diagnosis, tumor grade, stage, hormone receptor status, surgery, and radiotherapy (Table S3). The PS was then used as an adjustment factor in the Cox regression models. Survival curves and multivariate Cox regression with a competing risk model were used to compare differences in OS- and BC-specific survival between patients with CBC and patients with unilateral BC. Additional subgroup survival analysis was performed based on stage. 

All data were obtained using SEER*Stat Software version 8.3.4 [18]. Statistical analysis was performed using Statistical analysis system (SAS) version 9.4. Joinpoint regression analysis was performed using Joinpoint software version 4.5.0.1 [19]. All *P*-values were two-sided; *P* ≤ 0.05 was considered significant.

## 3. Results

### 3.1. Occurrence Pattern of CBC Incidence

**Figure 1:**
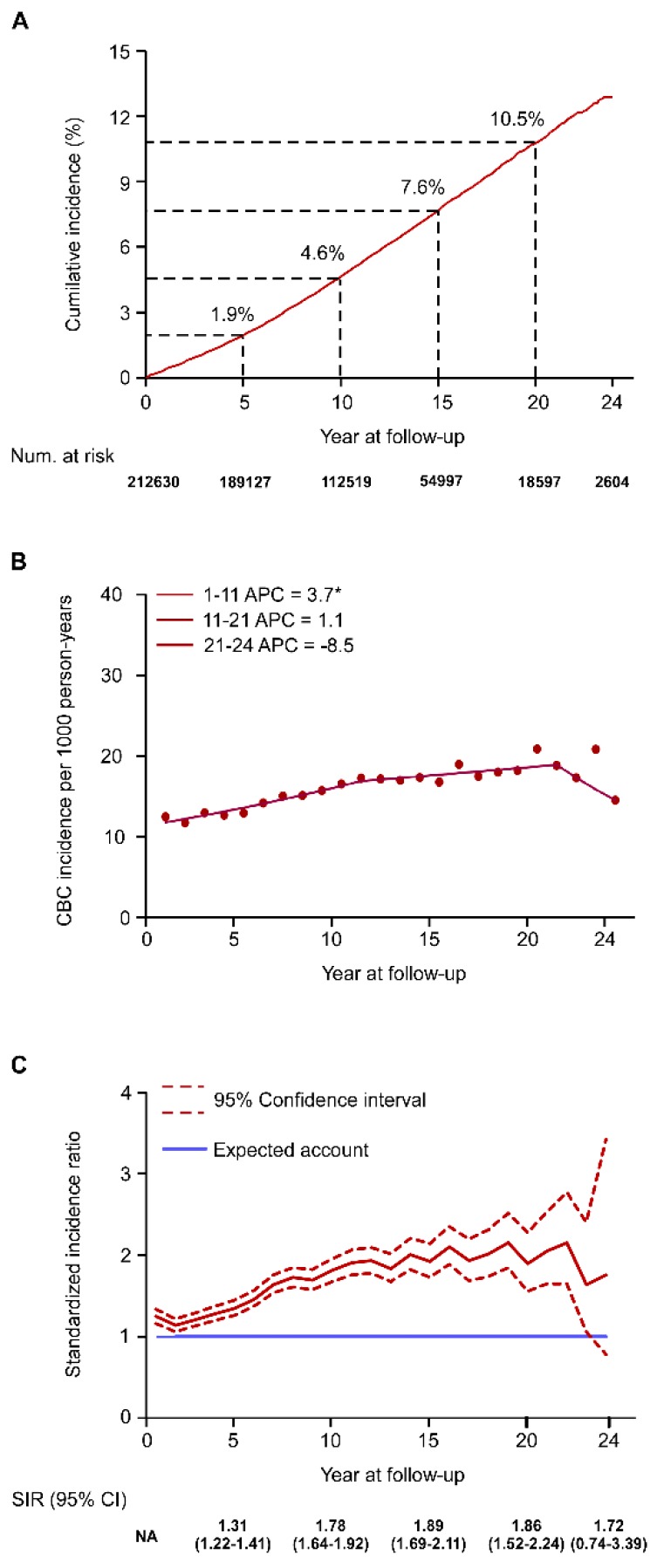
Temporal trend in contralateral breast cancer (CBC) incidence over the 24-year follow-up period (1990–2013). (**A**) Cumulative incidence of CBC in early-stage breast cancer (BC). (**B**) Trend of CBC incidence per 1000 person-years estimated by joinpoint regression (Trend 1: years 1–11, Trend 2: years 11–21, Trend 3: years 21–24). (**C**) Trend in standardized incidence ratio (SIR) CBC in early-stage BC compared with the general population. * *P* < 0.05. NA = not available.

A total of 212,630 patients were diagnosed with early-stage BC, and 10,944 (5.1%) developed CBC in 1990–2009. The 5-, 10-, 15-, and 20-year cumulative incidences of CBC were 1.9, 4.6, 7.6, and 10.5%, respectively (Figure 1A). We examined the effect of patient and tumor characteristics on the risk of CBC by adjusting for the potential confounding factors (Table 1). Initial cancer diagnosis at a younger age (<40 years), black ethnicity, receiving radiotherapy for the first BC, and having hormone receptor-negative BC were significantly correlated with higher risk of CBC (Figure S1A–D and Table 1).

During the 24-year follow-up, the trend in CBC incidence had three phases (Figure 1B and Table 2). CBC incidence increased continuously in the first 11 years after the initial cancer diagnosis (Trend 1, APC = 3.7, 95% confidence interval (95% CI) = 2.9–4.5). The upward trend slowed at year 11–21 (Trend 2, APC = 1.1; 95% CI = −0.4 to 2.7), and the incidence tended to decline at year 21–24 (Trend 3, APC = −8.5; 95% CI = −24 to 10.1). Subgroup analysis based on age showed that CBC incidence in younger patients increased more rapidly than in older patients (Figure S2A and Table 2). Among patients aged ≥70 years, a significantly declining trend in CBC risk was observed between year 9 and 23 (Trend 2, APC = −4.3, 95% CI = −8.2 to −0.2; Figure S2A and Table 2). Regarding hormone receptor status, the increased trend in CBC in patients with initial hormone receptor-positive BC (Trend 1, APC = 2.3, 95% CI = −7.2 to 12.9; Trend 2, APC = 8.4, 95% CI = 6.9–9.8) was delayed compared with that in patients with initial hormone receptor-negative BC (Trend 1, APC = 44.1, 95% CI = 7–94; Trend 2, APC = 1.5, 95% CI = 0.2–2.7; Figure S2B and Table 2). Among patients who had undergone radiotherapy, the incidence of CBC increased continuously during follow-up, whereas no significant trend was observed in patients who had not undergone radiotherapy (Figure S2C and Table 2).

Furthermore, we assessed the temporal trend in excess risk of BC in BC patients compared to the risk in the general population. In the first 23 years of follow-up, the risk of secondary BC in BC patients was significantly higher than the risk of BC in the general population. The 5-, 10-, 15-, and 20-year SIR (95% CI) were 1.31 (1.22–1.41), 1.78 (1.64–1.92), 1.89 (1.69–2.11), and 1.86 (1.52–2.24), respectively (Figure 1C). At year 24, no significant excess morbidity risk was observed in the patients with BC (24-year SIR (95% CI), 1.72 (0.74–3.39)).

### 3.2. Overall and Cancer-Specific Survival

**Figure 2:**
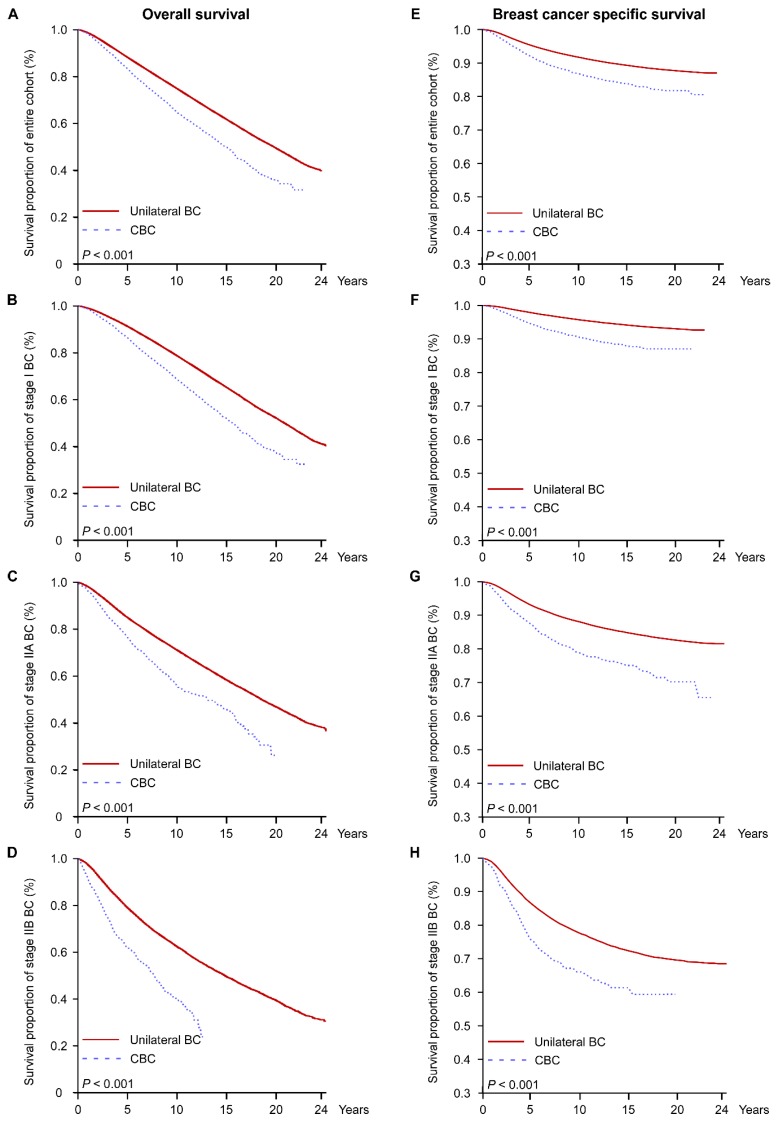
Overall and BC-specific survival analyses adjusted using the propensity scoring (PS) model between patients with early-stage unilateral BC and patients with early-stage CBC. All values are based on the PS model. Left - overall survival (OS): (**A**) entire cohort, (**B**) stage I, (**C**) stage IIA, and (**D**) stage IIB. Right—BC-specific survival: (**E**) entire cohort, (**F**) stage I, (**G**) stage IIA, and (**H**) stage IIB. BC = breast cancer; CBC = contralateral breast cancer.

The outcomes of patients with secondary BC are seldom reported. In patients with CBC and patients with unilateral BC, BC caused 36.1% and 27.9% of deaths, respectively (Table S2). Survival analysis was performed on patients with CBC and patients with unilateral BC. Both the multivariate-adjusted and PS-adjusted models showed that patients with CBC had significantly shorter OS than those with unilateral BC (unilateral BC vs. CBC hazard ratio (HR) = 0.776, 95% CI = 0.743–0.810; HR = 0.815, 95% CI = 0.780–0.852 for multivariate-adjusted and PS-adjusted, respectively; Figure 2A and Table 3). Subgroup survival analysis based on stage showed that patients with unilateral BC had better OS compared with the patients with CBC (Figure 2B–D and Table 3).

Although patients with CBC had worse OS, only a small proportion died due to BC. Accordingly, we analyzed BC-specific survival using a competing risk model. In patients with stage IIB CBC, 62.6% of patient deaths were due to BC, whereas 48.2% and 28.6% of deaths were BC-related in patients with stage IIA and stage I CBC, respectively (Table S2). In the entire cohort, patients with unilateral BC had 0.51 times lower BC-specific risk of mortality than patients with CBC (unilateral BC vs. CBC, HR = 0.482, 95% CI = 0.447–0.520; HR = 0.51, 95% CI = 0.474–0.549 for multivariate-adjusted and PS-adjusted, respectively; Figure 2E and Table 3). Moreover, CBC was correlated with worse BC-specific survival regardless of tumor stage (Figure 2F–H and Table 3).

## 4. Discussion

First, our study revealed the temporal trend in CBC incidence and identified the risk factors of CBC in patients with BC. Further, we compared the outcomes of patients with CBC with those of patients with unilateral BC, and found that the diagnosis of CBC adversely affected survival in early-stage BC.

The risk of CBC is greater in patients with BC than the risk of BC in the general population [20]. As cancer mortality is decreasing and life expectancy is increasing, patients with BC, especially those with early-stage BC, have an increased probability of developing secondary BC. In our study, we excluded patients with poor prognosis and short survival, including patients with local advanced or initial stage IV BC, to avoid underestimating the CBC incidence. We found that patients with BC have a significantly higher risk of CBC than the general population’s risk of BC, and that the excess risk remains significant until 23 years after the initial diagnosis of BC. Therefore, the purpose of surveilling patients with BC is not only to detect cancer recurrence but also to discover CBC development. Presently, most patients undergo physical and imaging examination one to four times per year for the first five years, and then once annually [21]. Patients should be more vigilant about examinations for CBC when CBC incidence is high, whereas excessive imaging would only increase medical costs and does not aid in the early detection of CBC. Studies on the variation trend of CBC incidence are needed to guide the management of cancer surveillance. We observed that CBC incidence increased continuously within the 11 years after the first diagnosis of BC, and remained at a peak in years 11 to 21. These results suggest that patients with BC have a high risk of CBC for over 21 years; therefore, they should attach great importance to the occurrence of CBC.

CBC incidence varies from person to person, and identifying the high-risk population for CBC is important for optimal surveillance and management of patients with BC. The risk of CBC decreases with increased age at initial diagnosis of BC [22,23]. Compared with older patients, younger patients tend to have unfavorable genetic profiles (including *BRCA1* and *BRCA2* mutation), which may contribute to a high risk of BC [24,25,26]. Consistently, we found that younger patients with BC (<40 years) had significantly higher CBC risk than older patients. Moreover, we found that younger patients reached peak CBC morbidity earlier and that the peak lasted longer compared with older patients. As patients with cancer diagnosis at a younger age have a longer life expectancy than the older patients, shorter intervals between examination and a prolonged period of close follow-up should be considered. In addition, hormone receptor status was significantly correlated with risk of CBC [5,27]. In our study, patients with hormone receptor-negative BC were more susceptible to CBC. We assumed that the lower CBC risk was attributed to the wide use of endocrine treatment in hormone receptor-positive BC. Given the effectiveness of endocrine treatment, including aromatase inhibitors and tamoxifen, in BC prevention, most patients with hormone receptor-positive BC would undergo endocrine treatment in the first five years to prevent cancer recurrence, which simultaneously lowers their risk of CBC [28,29,30]. Conversely, patients with hormone receptor-negative BC respond poorly to endocrine treatment, so that most refuse endocrine treatment and are more likely to develop CBC. Interestingly, we observed that the risk of CBC in hormone receptor-positive BC temporarily decreased in the first five years, and increased rapidly thereafter. These results indicate that the protective effect of endocrine treatment against CBC is transitory and that patients who undergo endocrine therapy should remain vigilant upon completing endocrine treatment. As the duration of endocrine treatment has been extended to 10 years, whether the extended endocrine treatment can further delay CBC development in patients with hormone receptor-positive BC should be studied further [29,31]. Future studies on CBC risk should assess the protective effect of endocrine treatment between patients with five-year regimens and those with 10-year regimens.

In our study, we discovered that patients with CBC had significantly worse survival compared to patients with unilateral disease in early-stage BC. These findings are consistent with the previous studies that patients who developed CBC early, in the first five years after the initial diagnosis of BC, have excess mortality risk compared to patients with unilateral BC [2,4,14]. At present, no evidence exists that correlates CBC with more aggressive biological behavior compared to the first BC, and we found that the higher risk of mortality remained significant in different BC stages (stages I, IIA, and IIIB). Therefore, we assume that the poor prognosis in patients with CBC might be attributed to the influence of cancer treatment. Drug resistance induced by prior therapy and cumulative toxicity caused by chemotherapy may be reasons for the excess mortality risk in CBC, and over-dosage of radiation to normal organs caused by a second regimen of radiotherapy might also increase the risk of mortality. Although the reason patients with CBC have a worse outcome is unknown, our results indicate that CBC diagnosis is correlated with poor prognosis and that reducing CBC occurrence definitively improves BC survival. Patients with a high risk of CBC may consider prophylactic resection of the contralateral breast for preventing CBC.

Our study was based on SEER data, comprising patient information collected from nine registries from 1990 to 2009 in the United States. Therefore, the variation trend in CBC incidence we report is highly representative of the general population. However, our study has limitations. As this is a retrospective study, missing data and selection bias are inevitable. Thus, we used the PS model to adjust selection bias from tumor- and patient-specific variables. The details of systematic therapy, including endocrine treatment and chemotherapy, were not available, and the relevant biases may exist. 

## 5. Conclusions

In conclusion, our results showed that CBC incidence increased continuously in the first 11 years after the initial cancer diagnosis, and the upward trend slowed during years 11 to 21, and tended to decline at years 21 to 24. Furthermore, the diagnosis of CBC is correlated with poor prognosis regardless of tumor stage in early-stage BC. Consequently, patients should accept intensive surveillance for 20 or more years after the initial diagnosis of BC.

## Figures and Tables

**Table 1:** Univariate and multivariate Cox regression for the occurrence of contralateral breast cancer (CBC) in early stage breast cancer (BC) patients.

	Univariate			Multivariate ^a^		
Variable	HR	95% CI	*P* Value	HR	95% CI	*P* Value
**Age**						
<40	1			1		
40–49	0.708	0.656–0.763	<0.001	0.707	0.652–0.768	<0.001
50–59	0.706	0.656–0.760	<0.001	0.709	0.654–0.768	<0.001
60–69	0.747	0.694–0.805	<0.001	0.759	0.700–0.824	<0.001
≥70	0.703	0.652–0.758	<0.001	0.746	0.686–0.811	<0.001
**Race**						
Black	1			1		
White	0.739	0.693–0.788	<0.001	0.762	0.709–0.819	<0.001
Other	0.734	0.670–0.803	<0.001	0.744	0.674–0.822	<0.001
**Year of diagnosis**						
1990–1994	1			1		
1995–1999	1.062	1.011–1.115	0.016	1.038	0.983–1.095	0.181
2000–2004	1.04	0.985–1.097	0.158	0.994	0.937–1.055	0.85
2005–2009	0.867	0.808-0.930	<0.001	0.837	0.775–0.903	<0.001
**Stage**						
I	1			1		
IIA	0.928	0.889–0.969	0.01	0.91	0.868–0.954	<0.001
IIB	0.935	0.872–1.002	0.056	0.936	0.868–1.008	0.079
**Hormone receptor status**						
+ vs. −	0.814	0.775–0.855	<0.001	0.839	0.797–0.883	<0.001
**Radiotherapy**						
Yes vs. No/Refused	1.261	1.213–1.310	<0.001	1.258	1.206–1.312	<0.001
**Sex**						
Male vs. Female	0.332	0.209–0.526	<0.001	0.363	0.215–0.613	<0.001

^a^ Co-variates for multivariate adjustment: age, Hispanic origin, race, sex, marital status, year of diagnosis, tumor grade, stage, hormone receptor status, and RT. BC = breast cancer; CBC = contralateral breast cancer; RT = radiotherapy; HR = hazard ratio; 95% CI = 95% confidence interval.

**Table 2:** Jointpoint regression for CBC incidence in patients with early stage BC.

Characteristic	Total					
	Trend 1		Trend 2		Trend 3	
	Period	APC (95% CI)	Period	APC (95% CI)	Period	APC (95% CI)
**Total**	1–11	3.7 *, (2.9–4.5)	11–21	1.1, ((−0.4)–2.7)	21–24	−8.5 ((−24)–10.1)
**Age**						
<40	1–4	51.4 *, (12.8–103.1)	4–24	0.6, ((−1.6)–3.0)	NA	NA
40–49	1–11	8.0 *, (6.1–10.0)	11–24	1.5, ((−0.8)–3.9)	NA	NA
50–59	1–12	8.5 *, (6.8–10.2)	12–24	1.2, ((−1.8)–4.4)	NA	NA
60–69	1–11	6.4 *, (4.8–8.0)	11–23	−0.2, ((−2.6)–2.4)	NA	NA
≥70	1–9	5.7 *, (3.0–8.5)	9–23	−4.3 *, ((−8.2)–(−0.2))	NA	NA
**Race**						
Black	1–7	17.7 *, (11.1–24.6)	7–23	−0.5, ((−3.0)–2.1)	NA	NA
White	1-10	7.1 *, (6.2–7.9)	10–24	1.0 *, (0.1–2.0)	NA	NA
Other	1-24	6.6 *, (5.2–8.0)	NA	NA	NA	NA
**Year of diagnosis**						
1990–1994	1–4	14.7 *, (2.8–28)	4–24	2.1 *, (1.3–2.8)	NA	NA
1995–1999	1–3	24.1, ((−2.2)–57.5)	3–19	4.2 *, (3.1–5.2)	NA	NA
2000–2004	1–14	6.7 *, (5.5–8.0)	NA	NA	NA	NA
2005–2009	1–3	–16.5, ((−43.4)–23.1)	3–9	21.0 *, (10.3–32.7)	NA	NA
**Stage**						
I	1–8	8.8 *, (7.6–10.0)	8–19	2.1 *, (1.2–3.0)	19–24	−5.6 *, ((−13.6)–3.2)
IIA	1–10	7.0 *, (5.2–8.9)	10–24	1.4, ((−0.6)–3.4)	NA	NA
IIB	1–23	5.4 *, (4.2–6.6)	NA	NA	NA	NA
**Hormone receptor status**						
+	1–3	2.3, ((−7.2)–12.9)	3–11	8.4 *, (6.9–9.8)	11–24	0.8, ((−0.4)–2.1)
–	1–3	44.1 *, (7.0–94.0)	3–24	1.5 *, (0.2–2.7)	NA	NA
**RT**						
Yes	1–4	18.5 *, (10.9–26.5)	4–11	8.0 *, (6.0–10.2)	11–24	2.9 *, (1.5–4.4)
No/Refused	1–4	−2.5, ((−9.8)–5.4)	4–7	10.2, ((−6.9)–30.4)	7–24	−0.3, ((−1.6)–1.0)

* *P* < 0.05. BC = breast cancer; CBC = contralateral breast cancer; RT = radiotherapy; APC = annual percent change; 95% CI = 95% confidence interval.

**Table 3:** Subgroup analysis of overall and breast cancer-specific survival (unilateral vs. contralateral BC) based on stage (I, IIA, and IIB).

Stage	Unadjusted ^a^		Multivariate-Adjusted ^a,b^		PS-Adjusted ^a,b^	
	HR (95% CI)	*P* Value	HR (95% CI)	*P* Value	HR (95% CI)	*P* Value
**Overall survival**						
All	0.676 (0.648–0.706)	<0.001	0.776 (0.743–0.810)	<0.001	0.815 (0.780–0.852)	<0.001
I	0.638 (0.606–0.673)	<0.001	0.777 (0.737–0.820)	<0.001	0.825 (0.782–0.870)	<0.001
IIA	0.627 (0.575–0.684)	<0.001	0.754 (0.691–0.823)	<0.001	0.790 (0.724–0.863)	<0.001
IIB	0.604 (0.520–0.702)	<0.001	0.750 (0.645–0.873)	<0.001	0.716 (0.616–0.833)	<0.001
**Breast cancer-specific survival**						
All	0.630 (0.586–0.678)	<0.001	0.482 (0.447–0.520)	<0.001	0.510 (0.474–0.549)	<0.001
I	0.457 (0.414–0.505)	<0.001	0.427 (0.385–0.474)	<0.001	0.445 (0.402–0.494)	<0.001
IIA	0.559 (0.492–0.634)	<0.001	0.521 (0.458–0.594)	<0.001	0.552 (0.486–0.627)	<0.001
IIB	0.609 (0.502–0.740)	<0.001	0.572 (0.467–0.701)	<0.001	0.594 (0.488–0.723)	<0.001

^a^ 4,786 patients with CBC were excluded in the survival analysis due to: (1) diagnosis of stage III, IV, or unknown stage and (2) time of diagnosis between 2010–2013. ^b^ Co-variates for multivariate and PS-adjustment: age, Hispanic origin, race, sex, marital status, year of diagnosis, tumor grade, stage, hormone receptor status, surgery, and RT. BC = breast cancer; CBC = contralateral breast cancer; RT = radiotherapy; PS = propensity score; HR = odds ratio; 95% CI = 95% confidence interval.

## Supplementary Materials

The following are available online at http://www.mdpi.com/2077-0383/7/6/133/s1, Figure S1. Estimates of CBC incidence among subgroups: (A) age, (B) race, (C) radiotherapy, and (D) hormone receptor status. RT = radiotherapy; Figure S2. Trends in CBC incidence per 1000 person-years estimated by joinpoint regression among subgroups: (A) age, (B) hormone receptor status, and (C) radiotherapy. CBC = contralateral breast cancer, RT+ = underwent radiotherapy, and RT− = did not undergo radiotherapy; Table S1: Characteristics of patients with unilateral or contralateral breast cancer; Table S2: Cause of death among patients with unilateral BC or CBC; Table S3: Comparison of characteristics between patients with unilateral BC and CBC, chi-square test.
