# Supplementary material for: Patterns of Occurrence and Outcomes of Contralateral Breast Cancer: Analysis of SEER Data
Source: J Clin Med. 2018 May 31;7(6):133. doi: 10.3390/jcm7060133 (PMC6025574; doi:10.3390/jcm7060133)
Supplement: Supplementary file 1 [file jcm-07-00133-s001.pdf]

Supplement method

SEER Treatment Variables and Covariates

Patients related variables included age of diagnosis (<40 years, 40-49 years, 50-59 years, 60-69 years and ≥70 years), year of cancer diagnosis(1990-1994, 1995-1999, 2000-2004, 2005-2009, and 2010-2013), marital status (married, unmarried/divorced and unknown), Hispanic origin (yes and no), race (white, black and other including American Indian/AK Native, Asian/Pacific Islander) and sex (female and male). Tumor related variables included tumor grade (grade I, grade II, grade III and unknown), stage (I, IIA, IIB, III, IV, and unknown) and Hormone receptor status (positive, negative and unknown). Treatment related variables included surgery (mastectomy, breast-conserving surgery, other and no/unknown) and radiotherapy (yes, refused, no, and unknown). Cause of death was stratified as breast cancer and other causes.

Table S1. Characteristics of patients with unilateral or contralateral breast cancer

Characteristic	Primary diagnosis of BC				CBC	
	No CBC		With CBC			
	Num	%	Num	%	Num	%
**Total**	201686	100	10944	100		
**Age group, y**						
<40	11096	5.5	953	8.7	272	2.5
40-49	37874	18.8	2282	20.9	1155	10.6
50-59	48256	23.9	2760	25.2	2202	20.1
60-69	45696	22.7	2636	24.1	2859	26.1
⩾70	58764	29.1	2313	21.1	4456	40.7
**Hispanic**						
Yes	9604	4.8	500	4.6	500	4.6
No	192082	95.2	10444	95.4	10444	95.4
**Marital status**						
Married	117029	58.0	6789	62.0	5702	52.1
Unmarried/Divorced	78610	39.0	3915	35.8	4555	41.6
Unknown	6047	3.0	240	2.2	687	6.3
**Year of diagnosis**						
1990-1994	41005	20.3	3399	31.1	423	3.9
1995-1999	49012	24.3	3553	32.5	1399	12.8
2000-2004	54986	27.3	2758	25.2	2461	22.5
2005-2009	56683	28.1	1234	11.3	3527	32.2
2010-2013	NA	NA	NA	NA	3134	28.6
**Race**						
White	168829	83.7	9053	82.7	9053	82.7
Black	16095	8.0	1038	9.5	1038	9.5
Other[a]	16128	8.0	849	7.8	849	7.8
Unknown	634	0.3	4	0.0	4	0.0
**Sex**						
Female	200418	99.4	10926	99.8	10926	99.8
Male	1268	0.6	18	0.2	18	0.2
**Tumor grade**						
Grade I	39501	19.6	2008	18.3	2310	21.1
Grade II	76979	38.2	3919	35.8	4179	38.2
Grade III	57897	28.7	3057	27.9	3237	29.6
Unknown	27309	13.5	1960	18.0	1218	11.1
**Stage**						
I	121557	60.3	7096	64.8	6320	57.7
IIA	59382	29.4	2940	26.9	1924	17.6
IIB	20747	10.3	908	8.3	512	4.7
III	NA	NA	NA	NA	755	6.9
IV	NA	NA	NA	NA	361	3.3

Unknown	NA	NA	NA	NA	1072	9.8
**Hormone receptor status**						
+	148174	73.5	7581	69.3	7663	70.0
-	32210	16.0	1990	18.2	2051	18.7
Unknown	21302	10.6	1373	12.5	1230	11.2
**Surgery**						
Mastectomy	76854	38.1	3369	30.8	6086	55.6
BCT	119495	59.2	7295	66.7	4076	37.2
Other	3457	1.7	215	2.0	77	0.7
No/Unknown	1880	0.9	65	0.6	705	6.4
**Radiotherapy**						
Yes	103625	51.4	6282	57.4	3163	28.9
Refused	1687	0.8	122	1.1	124	1.1
No	92427	45.8	4318	39.5	7424	67.8
Unknown	3947	2.0	222	2.0	233	2.1
**Status**						
Alive	134551	66.7	7480	68.3	7480	68.3
Dead breast	18750	9.3	1606	14.7	1606	14.7
Dead other	48385	24.0	1858	17.0	1858	17

Abbreviation: BC = breast cancer; CBC = contralateral breast cancer; BCT = breast-conserving therapy.

[a]Other including: American Indian/Alaska Native,Asian/Pacific Islander.

Table S2. Cause of death among patients with unilateral BC or CBC

Cause of death	Unilateral BC				CBC			
	Total No.(%)	Stage I No.(%)	Stage IIA No.(%)	Stage IIB No.(%)	Total No.(%)	Stage I No.(%)	Stage IIA No.(%)	Stage IIB No.(%)
Num	201686	121557	59382	20747	6158	4502	1315	341
Alive	134551 (66.7)	84610 (69.6)	38005 (64)	11936 (57.5)	4006 (65.1)	3049 (67.7)	790 (60.1)	167 (49)
**Cause of death, 100%**								
Breast cancer	18750 (27.9)	6200 (16.8)	7730 (36.2)	4820 (54.7)	777 (36.1)	415 (28.6)	253 (48.2)	109 (62.6)
Cardiovascular/Cerebrovascular disease	12994 (19.4)	8251 (22.3)	3692 (17.3)	1051 (11.9)	343 (15.9)	270 (18.6)	52 (9.9)	21 (12.1)
Pulmonary disease	2698 (4.0)	1772 (4.8)	739 (3.5)	187 (2.1)	91 (4.2)	76 (5.2)	11 (2.1)	4 (2.3)
Infectiouss disease	1772 (2.6)	1113 (3.0)	512 (2.4)	147 (1.7)	45 (2.1)	33 (2.3)	11 (2.1)	1 (0.6)
Diabetes mellitus	1404 (2.1)	824 (2.2)	432 (2.0)	148 (1.7)	35 (1.6)	24 (1.7)	9 (1.7)	2 (1.1)
Other	29517 (44.0)	18787 (50.8)	8272 (38.7)	2458 (27.9)	861 (40.1)	635 (43.7)	189 (36)	37 (21.3)

Abbreviation: BC = breast cancer; CBC = contralateral breast cancer.

Table S3. Comparison of characteristics between patients with unilateral BC and CBC, chi-square test

Characteristic	Unilateral BC		CBC		P value
	Num	%	Num	%	
**Total**	201686		10944		
**Selected**	201686	100	6158[a]	100	
**Age group, y**					
<40	11096	5.5	155	2.5	<0.001
40-49	37874	18.8	712	11.6	
50-59	48256	23.9	1287	20.9	
60-69	45696	22.7	1522	24.7	
⩾70	58764	29.1	2482	40.3	
**Hispanic origin**					
Yes	9604	4.8	255	4.1	0.024
No	192082	95.2	5903	95.9	
**Race**					
White	168829	83.7	5177	84.1	<0.001
Black	16095	8	521	8.5	
Other[b]	16128	8	456	7.4	
Unknown	634	0.3	4	0.1	
**Sex**					
Female	200418	99.4	6149	99.9	<0.001
Male	1268	0.6	9	0.1	
**Marital status**					
Married	117029	58.0	3280	53.3	<0.001
Unmarried/Divorced	78610	39.0	2586	42.0	
Unknown	6047	3.0	292	4.8	
**Year of diagnosis**					
1990-1994	41005	20.3	304	4.9	<0.001
1995-1999	49012	24.3	1037	16.8	
2000-2004	54986	27.3	1938	31.5	
2005-2009	56683	28.1	2879	46.8	
**Tumor grade**					
Grade I	39501	19.6	1416	23	<0.001
Grade II	76979	38.2	2365	38.4	
Grade III	57897	28.7	1805	29.3	
Unknown	27309	13.5	572	9.3	
**Stage**					
I	121557	60.3	4502	73.1	<0.001
IIA	59382	29.4	1315	21.4	
IIB	20747	10.3	341	5.5	
**Hormone receptor status**					
+	148174	73.5	4310	70	<0.001
-	32210	16	1201	19.5	
Unknown	21302	10.5	647	10.5	

**Surgery**					
Mastectomy	76854	38.1	3431	55.7	<0.001
BCT	119495	59.2	2583	41.9	
Other	3457	1.7	49	0.8	
No/Unknown	1880	0.9	95	1.6	
**RT**					
Yes	103625	51.4	1881	30.5	<0.001
Refused	1687	0.8	58	0.9	
No	92427	45.8	4122	66.9	
Unknown	3947	2	97	1.7	

Abbreviation: BC = breast cancer; CBC = contralateral breast cancer; BCT = breast-conserving therapy; RT = radiotherapy.

[a]4786 patients with CBC were excluded in the survival analysis due to: (1) diagnosis of stage III, IV or unknown stage and (2) time of diagnosis between 2010-2013.

[b]Other including: American Indian/AK Native,Asian/Pacific Islander.

Titles and legends to supplementary figures

Figure S1. Estimates of CBC incidence among subgroups: **A)** age, **B)** race, **C)** radiotherapy, and **D)** hormone receptor status. RT = radiotherapy.

Figure S2. Trends of CBC incidence per 1000 person-years estimated by joinpoint regression among subgroups: **A)** age, **B)** hormone receptor status, **C)** radiotherapy. CBC = contralateral breast cancer; RT+ = underwent radiotherapy; RT- = did not undergo radiotherapy.

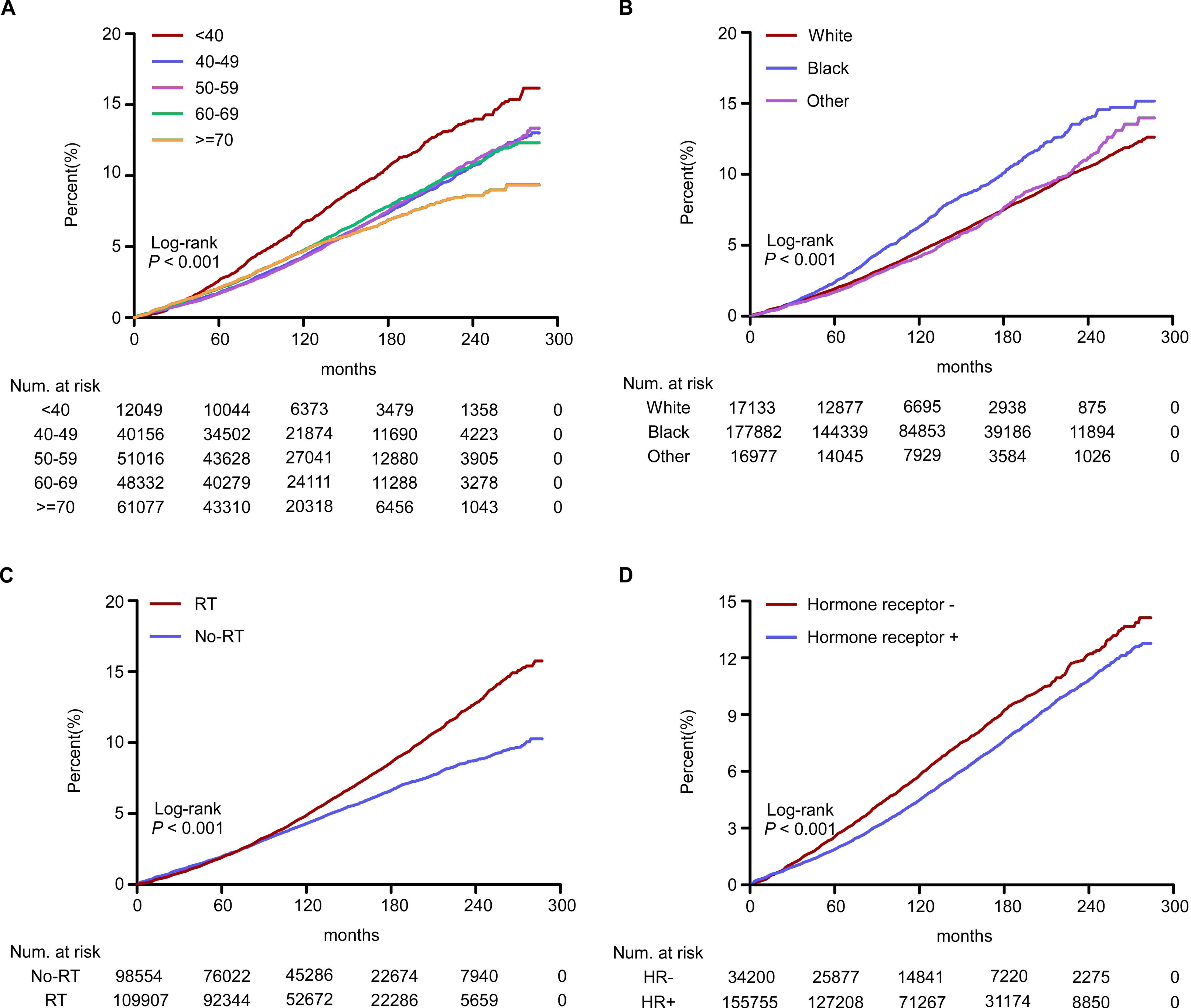
A
<40
40-49
50-59
60-69
>=70
Percent(%)
Log-rank
P < 0.001
months
Num. at risk
<40 12049 10044 6373 3479 1358 0
40-49 40156 34502 21874 11690 4223 0
50-59 51016 43628 27041 12880 3905 0
60-69 48332 40279 24111 11288 3278 0
>=70 61077 43310 20318 6456 1043 0
B
White
Black
Other
Percent(%)
Log-rank
P < 0.001
months
Num. at risk
White 17133 12877 6695 2938 875 0
Black 177882 144339 84853 39186 11894 0
Other 16977 14045 7929 3584 1026 0
C
RT
No-RT
Percent(%)
Log-rank
P < 0.001
months
Num. at risk
No-RT 98554 76022 45286 22674 7940 0
RT 109907 92344 52672 22286 5659 0
D
Hormone receptor -
Hormone receptor +
Percent(%)
Log-rank
P < 0.001
months
Num. at risk
HR- 34200 25877 14841 7220 2275 0
HR+ 155755 127208 71267 31174 8850 0

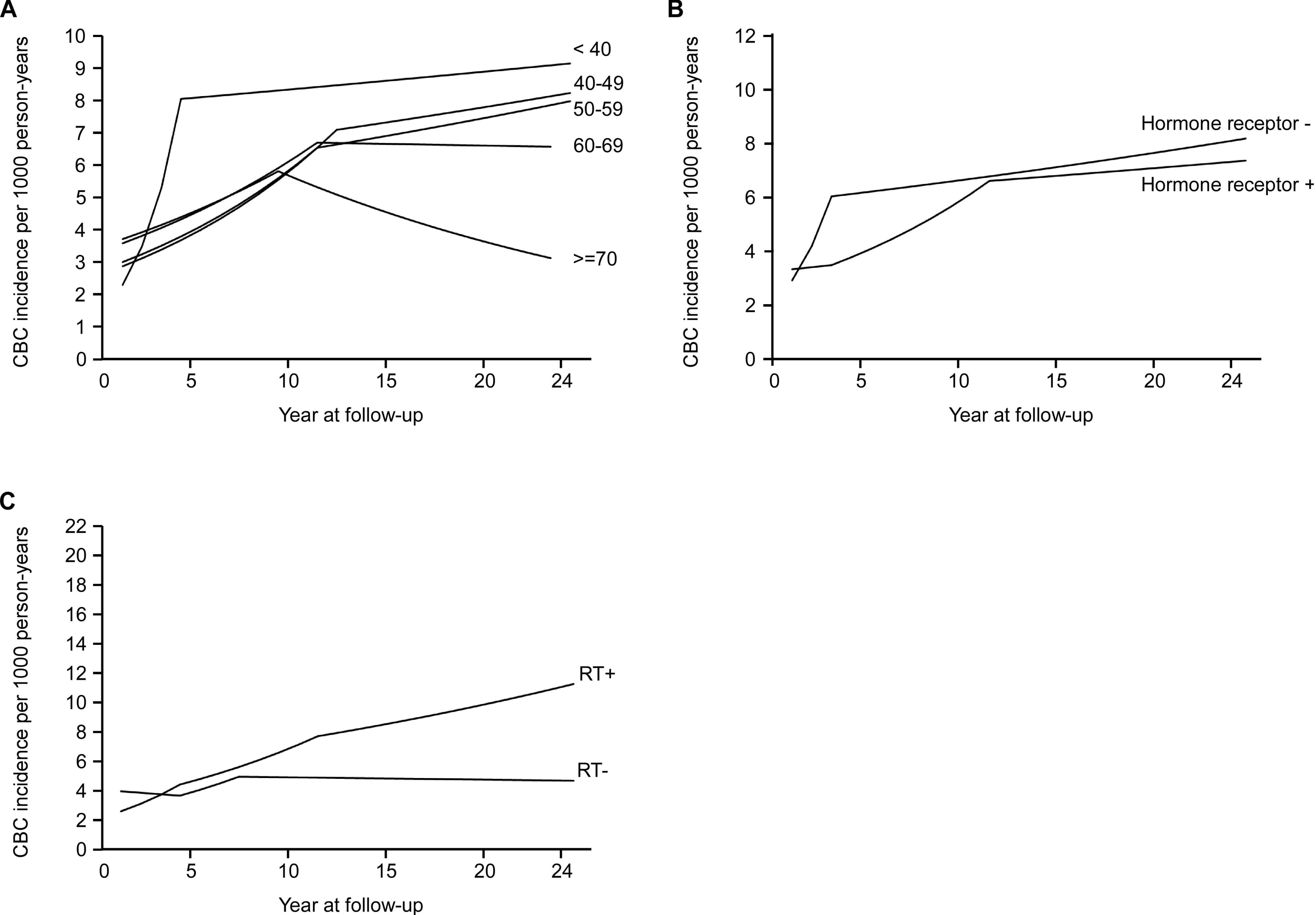
A
CBC incidence per 1000 person-years
Year at follow-up
< 40
40-49
50-59
60-69
>=70
B
Hormone receptor -
Hormone receptor +
C
RT+
RT-
